# High Prevalence of Self-Reported Undiagnosed HIV despite High Coverage of HIV Testing: A Cross-Sectional Population Based Sero-Survey in South Africa

**DOI:** 10.1371/journal.pone.0025244

**Published:** 2011-09-28

**Authors:** Katharina Kranzer, Nienke van Schaik, Unice Karmue, Keren Middelkoop, Elaine Sebastian, Stephen D. Lawn, Robin Wood, Linda-Gail Bekker

**Affiliations:** 1 Department of Clinical Research Unit, London School of Hygiene and Tropical Medicine, London, United Kingdom; 2 The Desmond Tutu HIV Centre, University of Cape Town, Cape Town, South Africa; 3 Northeastern University in Boston, Boston, Massachusetts, United States of America; 4 South African Centre for Epidemiological Modeling and Analysis, University of Stellenbosch, Stellenbosch, South Africa; Rush University, United States of America

## Abstract

**Objectives:**

To measure HIV prevalence and uptake of HIV counseling and testing (HCT) in a peri-urban South African community. To assess predictors for previous HIV testing and the association between the yield of previously undiagnosed HIV and time of last negative HIV test

**Methods:**

A random sample of 10% of the adult population (≥15 years) were invited to attend a mobile HCT service. Study procedures included a questionnaire, HIV testing and CD4 counts. Predictors for previous testing were determined using a binominal model.

**Results:**

1,144 (88.0%) of 1,300 randomly selected individuals participated in the study. 71.0% (68.3–73.6) had previously had an HIV test and 37.5% (34.6–40.5) had tested in the past 12 months. Men, migrants and older (>35 years) and younger (<20 years) individuals were less likely to have had a previous HIV test. Overall HIV prevalence was 22.7 (20.3–25.3) with peak prevalence of 41.8% (35.8–47.8) in women aged 25.1–35 years and 37.5% (26.7–48.3) in men aged 25.1–45 years. Prevalence of previously undiagnosed HIV was 10.3% (8.5–12.1) overall and 4.5% (2.3–6.6), 8.0% (CI 3.9–12.0) and 20.0% (13.2–26.8) in individuals who had their most recent HIV test within 1, 1–2 and more than 2 years prior to the survey.

**Conclusion:**

The high burden of undiagnosed HIV in individuals who had recently tested underscores the importance of frequent repeat testing at least annually. The high prevalence of previously undiagnosed HIV in individuals reporting a negative test in the 12 months preceding the survey indicates a very high incidence. Innovative prevention strategies are needed.

## Introduction

HIV counseling and testing (HCT) services are important entry points for prevention and care [1]. Studies from different countries have shown that individuals take precautions to protect their partners once they know they are HIV positive [2], [3], [4] and modeling studies have found HCT to offer substantial clinical benefits and to be cost-effective even in settings where linkage and access to care is limited [5].

The past decade has seen a rapid global scale-up of HCT [6]. Recent surveys from Tanzania, the Democratic Republic of the Congo, Kenya, Zambia, Swaziland and South Africa reported that between 8.6 and 56.6% of women and 9.2 and 43.0% of men ever had an HIV test [6], [7]. HCT uptake is associated with a range of socio-demographic factors, and is generally lower among men, younger and older age groups, those with limited education and income [6], [8], [9]. Identifying characteristics of individuals who have never tested is important to develop services targeted at first time testers and thus to achieve universal access to HCT.

Sexually active individuals in high HIV prevalence settings are at continuous risk of infection and should therefore test at regular intervals. The World Health Organization (WHO) recommends annual testing in high HIV prevalence settings as do the 2010 South African guidelines [10], [11]. A recent study from South Africa found annual screening to be very cost-effective even in the Western Cape, the province with the lowest rates of HIV infection in South Africa [5]. Despite the importance of annual testing, population surveys from six sub-Saharan African countries showed a median of only 19% of women and 10% of men had an HIV test in the 12 months preceding the survey [6]. Even though South Africa is above average with 24.7% of the population reporting a test within the last 12 months in 2008, it is still sub-optimal [7].

This study was conducted in a well characterized peri-urban community in the Western Cape, South Africa [12], [13]. The study community has been exposed to 9 years of community-based HIV prevention research and has seen provider-initiated HIV testing and antiretroviral therapy (ART) roll-out earlier than most other communities in South Africa. This community provides a unique opportunity to examine the effect of high HCT coverage and frequent testing. The aims of this study were to measure HIV prevalence and HCT uptake, to determine predictors for previous HIV testing and to assess the association between the yield of previously undiagnosed HIV and time of last negative test.

## Methods

### Ethics statement

Written informed consent was obtained from all individuals participating in the study. Data collection and analysis was approved by the University of Cape Town Ethics Committee and Partners Human Subjects Institutional Review Board and the London School of Hygiene and Tropical Medicine.

### Setting

The study was based in a peri-urban township in the greater area of Cape Town, South Africa. Regular household censuses have shown that the community has undergone a rapid population growth from 5000 residents in 1996 to 17000 in the most recent census in August 2010 [12]. Adult HIV prevalence was 23% in 2005 and 25% in 2008 as measured in previous population based HIV prevalence surveys.

The community was served by a single public-sector primary care clinic, which provided outpatient care including HCT and ART free of charge. A nearby hospital (5 km away) provided all secondary care, including inpatient and antenatal services. The hospital also provided ART for some HIV-infected individuals from the community. ART provision at the primary health care clinic and hospital began in 2004. Since 2005, there has been a significant scale-up of the ART program in this community, with 13% of all individuals infected with HIV receiving ART in 2005 and 21% in 2008 [14].

Voluntary counseling and testing services have been available to all individuals accessing either the local clinic or the hospital since 2001 with provider-initiated testing routinely given to any patient accessing TB services whose HIV status was unknown; this was extended to all pregnant females accessing the hospital or clinic in 2002 and patients accessing STI services in 2007. HIV testing rates rose from 4% of the total population per year in 2001 to 20% in 2006 [15]. The total number of tests performed in the primary health care clinic or hospital among residents of this community was more than 10500 between January 2004 and March 2009 [16]. The community has also been served by a mobile HCT service 1–2 days per month since July 2008. The mobile HCT service has done more than 1000 tests in this community.

### Community-based cross-sectional survey

A population-based HIV sero-prevalence survey was conducted between September and December 2010. A house-to-house enumeration of the community in August 2010 provided a database of 12520 residents 15 years or older of whom 1300 residents were randomly selected for inclusion in the study (10% of the community). Simple random sampling was performed using Stata 11.0 (Stata Corp. LP, College Station, TX, United States of America). Each adult resident in the community had an equal chance of being selected for the survey. The census 2010 data were used as a sampling frame. Field workers invited the selected individuals to attend the mobile HIV testing service. Field workers visited households of selected individuals up to 5 times to encourage participation. No study procedures were performed in people's homes. Consent, questionnaires and HIV testing were performed at the mobile HIV testing service when a potential participant attended the service.

### Mobile HIV testing service

The mobile HIV testing service used in this study has been described elsewhere [17]. In brief, this nurse-run and counselor-supported unit provides free HCT services in combination with free screening for other chronic conditions (i.e. hypertension, diabetes and obesity) and TB. HIV testing is performed according to the Provincial Government of the Western Cape guidelines [18]. Whilst the South African guidelines for HIV testing recommend written informed consent, the mobile, community based nature of this service led to the agreement by local health authorities to allow verbal consent in clients voluntarily accessing this service since 2008. Individuals approaching the mobile services give verbal consent for HIV testing which is recorded on the consultation form.

The mobile testing service was parked in front of the primary school in the centre of the community. It operated on weekdays and weekends as well as after hours to ensure that individuals with regular work had an opportunity to participate.

Participants could choose one of three options to receive their result: i) to test and receive their HIV result together with screening for chronic diseases, ii) to provide blood and not receive their HIV result, but undergo screening for chronic diseases or iii) to only provide blood and not receive their HIV result. Individuals who consented to rapid HIV testing and tested positive were subsequently staged according to the WHO staging manual and underwent a point of care CD4 count test (Alere™Pima™ CD4 Analyser, Waltham, MA, USA) using venous blood samples. All participants were compensated for transport and time with ZAR 70 (approximately 9.6 US dollars) gift vouchers.

### Data collection and management

Age, sex, nationality, migration history and previous HIV testing experience were recorded via a short questionnaire. Data were double entered and verified in EpiData version 3.1.

For HIV testing experience this included asking whether they had tested for HIV before and whether this was <3 months ago, 3–6 months ago, 6–12 months ago, 1–2 years ago or >2 years ago. Where individuals had tested on the mobile clinic before, this information was available from their previous records accessed using a biometric system. Recent migrants were defined as individuals who had moved into this community from either within South Africa or from neighboring countries within the 3 years preceding the survey.

Individuals who tested HIV positive and chose to receive their result were asked as part of the questionnaire if they were aware of their positive sero-status. Individuals who were unaware of their positive sero-status underwent the routine procedure of the mobile testing service for newly diagnosed HIV positive individuals. These procedures included clinical staging, CD4 count testing, pregnancy tests for women, screening for sexually transmitted disease, referrals to primary health care clinics and targeted counseling. All newly diagnosed HIV positive individuals were called by their counselor 7 days after diagnoses to ensure that they received enough support to deal with the new diagnosis. All counselors were extremely experienced and as such able to confirm if an individual was unaware of their sero-status. Twelve individuals who initially said that they were unaware of their HIV positive sero-status admitted to the counselor that they had known their positive sero-status before. This information was used to amend the data. For patients who chose to test anonymously and tested positive (N = 16) no additional information could be collected by the counselors.

### Statistical analysis

All analyses were carried out using Stata version 11.0 (Stata Corp. LP, College Station, TX, United States of America). Proportions and confidence intervals were calculated for categorical variables, and medians and interquartile ranges for continuous variables. The proportion of individuals who tested for HIV within the last year was calculated using individuals at risk for testing as a denominator. Thus, the denominator excluded individuals who had tested HIV positive more than one year ago. The prevalence of newly diagnosed HIV in individuals who had tested before excluded individuals known to be HIV positive from the denominator.

Differences in proportions between study participants who had tested previously and study participants who had never tested were calculated using cross-tabulation and χ2 test.. Risk ratios investigating association between age, gender, nationality, migration and previous HIV testing were calculated using a binominal model. Differences in median CD4 counts in individuals newly diagnosed with HIV, known to be HIV positive but not on ART and individuals on ART was assessed using the Kruskal-Wallis test.

## Results

### Characteristics of the study population

Of 1300 individuals randomly selected from the community, 1144 (88.0%) participated. Among the 156 individuals who did not participate in the study two had died before the study started, five refused to participate, and the remaining 149 did not attend the mobile HCT service despite multiple visits to their households. Individuals who did not participate in the study were older (median age 31; IQR [interquartile range] 27–38) and more likely to be men (76.2%) compared to individuals who participated in the study (median age 28; IQR 23–35, 48.6% men) (table 1).

**Table 1:** Characteristics, HCT coverage and HIV prevalence (N = 1144).

Variables	N	Percent	95% CI
**Characteristics of participants**			
Testing and receiving result	1078	94.2	92.9; 95.6
Women	588	51.4	48.5; 54.3
Age <20 years	134	11.7	9.9; 13.7
Age 20–34.9 years	714	62.4	59.5; 65.2
Age ≥35 years	296	25.9	23.4; 28.5
South African	1034	90.4	88.7; 92.1
Moved into the community during the past 3 years	309	27.2	24.6; 29.8
**Previous HIV testing**			
Previously tested for HIV	812	71.0	68.3; 73.6
Tested within the last year	386	37.5	34.6; 40.5
**HIV prevalence**			
Newly diagnosed HIV+	118	10.3	8.6; 12.1
Known HIV+	142	12.4	10.5; 14.3
HIV-	884	77.3	74.8; 79.7

The majority of study participants were South African and approximately one quarter had migrated to the study community within the last 3 years. Most migrants came from a neighboring province, the Eastern Cape, (52.6%) while 11.9% came from elsewhere in the Western Cape and 22.6% from neighboring countries. Non-South Africans (77.1%) were more likely to have recently migrated to the study community compared to South Africans (32.3%).

### Prevalence and predictors of previous HIV testing

71.0% (95% CI 68.3–73.6) of study participants had previously had an HIV test and more than one third (37.5%) had tested in the 12 months preceding the survey (table 1). The proportions of women, South Africans and long term residents were higher among individuals who had previously tested for HIV than among individuals who had never tested (table 2). In multivariate analysis women and South African nationals were more likely to have a previous HIV test. Migrants and younger and older individuals were less likely to have been tested before.

**Table 2:** Comparison of previously tested and untested individuals (N = 1144).

Variables	Previously tested for HIV (N = 812)			Never tested for HIV (N = 332)			p value (χ^2^ test)	Predictors of previous HIV test		
	N	Percent	95% CI	N	Percent	95% CI		RR	95% CI	p value
Women	486	59.9	56.5; 63.2	102	30.7	25.7; 35.7	<0.01	1.33	1.33; .143	<0.01
Age <20 years	75	9.2	7.3; 11.4	59	17.8	13.8; 22.2	<0.01	0.79	0.68; 0.91	<0.01
Age 20–34.9 years	532	65.5	62.1; 68.8	182	54.8	49.3; 60.3		1.00		
Age ≥35 years	205	24.3	22.3; 28.4	91	27.4	22.7; 32.5		0.87	0.81; 0.95	<0.01
South African	758	93.3	91.6; 95.1	276	83.1	79.1; 87.2	<0.01	1.29	1.06; 1.57	0.01
Moved into the community during the past 3 years	187	23.1	20.2; 26,1	122	37.2	31.9; 42.5	<0.01	0.83	0.80; 0.95	0.02

### HIV prevalence

Overall the proportion of people tested who agreed to receive their result was high (94.2%, 95%CI 92.9–95.6). A total of 66 individuals chose to test anonymously among whom 16 (24.2%) tested positive.

Overall HIV prevalence was 22.7% (95%CI 20.3–25.3). Just over half (54.6%, 95%CI 48.3–60.8) of the HIV-infected individuals knew their serostatus (table 1). Among the 142 HIV infected individuals who knew their positive serostatus, 87 were on ART (61.3%, 95%CI 52.7–69.3). The median CD4 count was 389 cells/uL (IQR 269–611) in individuals newly diagnosed with HIV, 430 cells/uL (IQR 287–631) in individuals known to be HIV positive but not on ART and 440 cells/uL (IQR 295–627) in individuals on ART. CD4 counts were not significantly different across the three groups.

**Figure 1:**
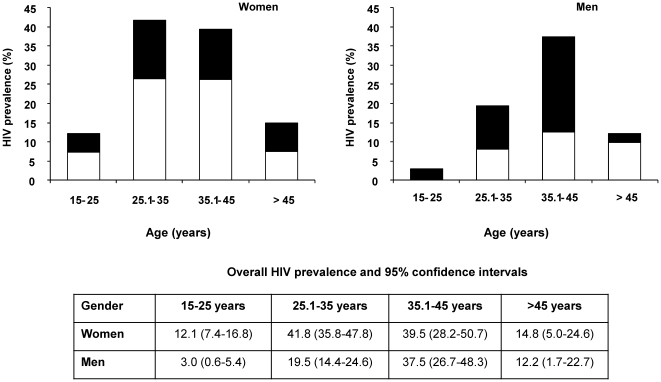
HIV prevalence by age and sex.

HIV prevalence and the proportion of undiagnosed HIV was associated with age and sex (figure 1). HIV prevalence was 12.1% (95%CI 7.4–16.8) in women 15–25 years of age compared to 41.8% (95%CI 35.8–47.8) in women aged 25.1–35. HIV prevalence in men was highest among the 35.1–45 year olds (37.5%, 95% 26.7–48.3). The proportion of positive tests that were new HIV diagnoses was significantly higher in men (62.1%, 95%CI 51.0–72.3) compared to women (37.0%; 95%CI 29.8–44.7).

### Prevalence of previously undiagnosed HIV

Prevalence of previously undiagnosed HIV was 18.4% (95%CI 14.2–22.4) in individuals who had never tested for HIV and 8.5% (95%CI 6.4–10.6) in individuals who reported HIV testing prior to the survey (p<0.001). Prevalence of previously undiagnosed HIV was 4.5% (95%CI 2.3–6.6), 8.0% (95%CI 3.9–12.0) and 20.0% (95%CI 13.2–26.8) in individuals who had their most recent HIV test within 1 year, 1–2 years and more than 2 years prior to the survey. There was no difference in prevalence in individuals last tested <3 (4.1%), 3–6 (4.9%), 6–12 (4.9%) months prior to the survey. A sensitivity analysis excluding the 16 individuals who tested positive but did not want to receive their test results revealed similar prevalence estimates except for the prevalence estimate in individuals last tested <3 months ago (1.2%).

In long-term residents, prevalence of previously undiagnosed HIV was 3.9% among those tested within 12 months and 6% among those tested within 1–2 years prior to the survey . Individuals who had recently moved into the community had a higher prevalence of previously undiagnosed HIV: 6.1% in individuals tested in the past 12 month and 14.3% in individuals tested 1–2 years ago.

## Discussion

This cross-sectional population based sero-survey found a peak HIV prevalence in the age group 25.1–35 in women (41.8%) and 35.1–45 in men (37.5%) in this peri-urban community. Almost half (45.4%) of the individuals infected with HIV were unaware of their HIV positive sero-status despite 71.0% of the population reporting that they had previously had an HIV test. Younger and older individuals, immigrants, individuals who had recently moved to the community and men were less likely to have previously tested for HIV. The prevalence of undiagnosed HIV was strongly associated with a history of HIV testing. Even among individuals who reported their most recent negative HIV test in the 12 months prior to the survey, the prevalence was 4.5%. CD4 count distributions were similar in HIV positive individuals on ART and not ART probably due to high ART coverage in this community [19].

In this community 71.0% had previously tested for HIV and 37.5% had an HIV test within the last 12 months. This is substantially higher than the corresponding national estimates from 2008 of 50.8% and 24.7%, respectively [7]. HCT services were strengthened and provider-initiated as well as voluntary testing was implemented as early as 2002 through the ongoing research program in this community which might explain some of the differences. Furthermore HCT has been scaled up on a national and provincial level over the last years. [7]. In April 2010 a national HCT campaign was launched aiming to test 15 million people for HIV by June 2011. This might have led to increased testing rates in the months before this survey.

In a community with such high testing rates one would expect the majority of HIV infected individuals to be aware of their HIV positive sero-status. The high proportion (45.6%) of undiagnosed HIV in this community may be due to a very high incidence. This study was not designed to measure HIV incidence. However a 4.5% prevalence of undiagnosed HIV in individuals reporting a negative test within the last 12 months translates into an incidence of 12.4 per 100 person-years assuming that the infection occurred at the mid-point between the last negative test and the positive test. Even when excluding individuals who tested positive, but did not want to receive their results, as these individuals might have known their positive sero-status before, the HIV incidence remains at 8.5 per 100 person-years. The method used to calculate these incidence estimates has not been validated and thus the estimates should be viewed with caution. They are, however in keeping with the incidence of 7.2 per 100 person-years reported from a cohort in this community in 2004–2005 [20]. Similar incidence rates of 6.4 per 100 person-years have been reported in women in rural and urban in KwaZulu Natal, South Africa [21]. These incident rates are in stark contrast to a recent estimate of 1.3 per 100 person-years using data from three national surveys [22]. National incidence estimates provide an average of incidence estimates across South Africa therefore very high incidence in some communities [23] might be compensated for by low incidence in others. In addition, it is well recognized that the South African HIV epidemic is heterogeneous with wide inter- and intra- provincial variation in HIV prevalence and incidence rates.

Individuals tested more recently are a self-selected group who might be at higher risk of HIV infection, which might bias the HIV incidence estimate. With almost a third of the population reporting that they had moved to the community in the last 3 years incidence might be overestimated due to high risk of HIV infection in migrants [24], [25]. Restricting the analysis to long-term residents only, reveals an HIV incidence estimate of 6.1/100 person-years, which is still extremely high. The HIV prevalence of 12.1% among young women provides further evidence for a very high HIV incidence in this community.

The high incidence in this community and the high prevalence of HIV in women aged 25.1–45 show that current prevention efforts – even in a setting where HIV prevention research is conducted – are failing. HIV prevalence estimates from this community with a peak prevalence of 41.8% in women and 37.5% in men are as high as reported from rural Kwazulu Natal, the South African province hardest hit by the HIV epidemic [26]. This community was exposed to more intensive prevention messages and better resourced HIV services than most other South African communities as evidenced by higher HCT coverage and the lower prevalence of newly diagnosed HIV in repeat testers in long-term residents as compared to recent migrants.

However prevention tools in 2011 are still very limited and these data would indicate that testing and awareness alone are insufficient to reduce HIV acquisition risk. Of note, a high HIV incidence has also been reported from the CAPRISA 004 microbicide trial in KwaZulu Natal, South Africa. Women participating in the CAPRISA 004 trial were all exposed to a package of prevention consisting of condoms, monthly testing and risk reduction counseling, but even so HIV incidence was reported at 9 per 100 person-years in the placebo assigned study group [27]. Clearly there is a need for additional and innovative prevention programs to reduce HIV incidence.

The high prevalence of undiagnosed HIV even in individuals who reported testing negative within the 3 months preceding the survey underscores the importance of counseling individuals on the window period as well as frequent repeated HIV testing especially for those at high risk of HIV infection. However, another reason for the high prevalence of undiagnosed HIV despite recent testing might be the low sensitivity of rapid HIV tests due to poor adherence with correct testing procedures in routine clinical practice and previous testing in the ‘window period’ during serocoversion [28].

Any annual screening program for a chronic and possibly fatal disease using a cheap point of care rapid test with a yield of 4.5% should be cost-effective [5]. With a yield of 4.5% in individuals who had tested negative in the 6 months preceding the survey even more frequent testing might be justified.

Previous testing experience and awareness of the HIV positive sero-status was assessed by self report which might be influenced by social desirability bias. In addition the exact time of testing might have been influenced by recall bias resulting in misclassification. Some of the individuals participating in the survey had tested at the mobile clinic before (N = 50). All but two reported the correct time of previous test. Bias and chance could explain the steady prevalence of 4–5% in individuals tested within 0–3 months, 3–6 months and 6–12 months prior to the survey. However an alternative explanation is that individuals testing at higher frequency might have a higher risk of HIV infection or that anonymous testers who tested positive in this survey knew their status already. Excluding those individuals did not change the overall results.

This study found that men, non-South Africans, younger and older individuals and individuals who had moved to the community within the last 3 years were less likely to have ever tested before, consistent with other studies from South Africa [7], [25], [29], [30]. More importantly the yield of newly diagnosed HIV was twice as high in individuals who had never tested before compared to individuals who reported a prior HIV test, emphasizing the need for frequent testing and expanding services to segments of the population which are hard to reach. This study highlights again that men are particularly underserviced as almost two thirds of HIV infected men were unaware of their HIV positive sero-status.

Among the limitations of this study are: a non-attendance rate of 12%. Reasons for non-attendance were temporary absenteeism (prolonged visits to the neighboring province), work commitment and silent refusals. These data are similar to other population based HIV sero-prevalence surveys from sub-Saharan Africa reporting absenteeism rates of 0.8–35.2% and refusal rates of 2.7–35.9% [31], [32], [33]. HIV prevalence found in this survey is consistent with estimates from previous surveys from the same community [34], thus non-response bias due to differences in age and gender between attendees and non-attendees seems negligible.

Fear of stigma and lack of confidentiality have been shown to be a major barrier for HIV testing [35], [36], [37], [38], [39], [40]. The high uptake of open (non-anonymous testing) is particularly encouraging and might be attributed to a well functioning and efficient ART program, reduced stigma due to a long period (9 years) of community-based HIV prevention research in this community and the fact that none of the team members working on the mobile HCT service were part of the community.

In conclusion this study showed a high burden of undiagnosed HIV despite high HCT coverage. The yield of previously undiagnosed HIV was 4.5% in individuals with a negative HIV test within 12 months preceding the survey. This suggests a very high HIV incidence. The results emphasize the importance of repeat testing perhaps even more frequently than annually. It underscores the notion that innovative and effective prevention interventions in addition to post test counseling are urgently required.
